# Hypoxia-inducible factor-1 alpha, in association with inflammation, angiogenesis and MYC, is a critical prognostic factor in patients with HCC after surgery

**DOI:** 10.1186/1471-2407-9-418

**Published:** 2009-12-01

**Authors:** Chen-Xin Dai, Qiang Gao, Shuang-Jian Qiu, Min-Jie Ju, Ming-Yan Cai, Yong-Feng Xu, Jian Zhou, Bo-Heng Zhang, Jia Fan

**Affiliations:** 1 Liver Cancer Institute, Zhongshan Hospital and Shanghai Medical School, Fudan University, Shanghai, PR China; 2 Institute of Biomedical Sciences, Fudan University, Shanghai, PR China; 3 Center for Evidence Based Medicine, Fudan University, Shanghai, PR China

## Abstract

**Background:**

Despite well-studied tumor hypoxia in laboratory, little is known about the association with other pathophysiological events in the clinical view. We investigated the prognostic value of hypoxia-inducible factor-1 alpha (HIF-1alpha) in hepatocellular carcinoma (HCC), and its correlations with inflammation, angiogenesis and MYC oncogene.

**Methods:**

In a random series of 110 HCC patients, the mRNA of HIF-1alpha, inflammation related factors (COX-2, MMP7 and MMP9), angiogenesis related factors (VEGF and PDGFRA) and MYC in tumor tissue were detected by real-time RT-PCR and HIF-1alpha protein was assessed by immunohistochemistry. The correlations between HIF-1alpha mRNA and the factors mentioned previously, the relationship between HIF-1alpha and clinicopathologic features, and the prognostic value were analyzed.

**Results:**

The expression of both HIF-1alpha mRNA and protein in HCC were independent prognostic factors for overall survival (OS) (*P *= 0.012 and *P *= 0.021, respectively) and disease-free survival (DFS) (*P *= 0.004 and *P *= 0.007, respectively) as well. Besides, the high expression of HIF-1alpha mRNA and protein proposed an advanced BCLC stage and more incidence of vascular invasion. The mRNA of HIF-1alpha had significantly positive correlations to that of COX-2, PDGFRA, MMP7, MMP9, MYC, except VEGF. In addition to HIF-1alpha, COX-2 and PDGFRA were also independent prognosticators for OS (*P *= 0.004 and *P *= 0.010, respectively) and DFS (*P *= 0.010 and *P *= 0.038, respectively).

**Conclusion:**

HIF-1alpha in HCC plays an important role in predicting patient outcome. It may influence HCC biological behaviors and affect the tumor inflammation, angiogenesis and act in concert with the oncogene MYC. Attaching importance to HIF-1alpha in HCC may improve the prognostic and therapeutic technique.

## Background

**HEPATOCELLULAR **carcinoma (HCC) is the sixth most common cancer and the third most common cause of death from cancer worldwide, with increasing incidence in western countries [1]. Surgical resection provides an opportunity for cure, but the outcome remains dismal due to frequent tumor recurrence. Recently, better appreciations of the role that tumor microenvironment plays in tumor progression have bring a paradigm shift in developing strategies for cancer management. As a consequence, renewed emphasis has been placed on key features of the tumor microenvironment, in particular hypoxia, leading us to explore hypoxia related parameters for more accurate classification of this fetal disease.

As is well-recognized, hypoxia is a common mechanism in HCC as the solid tumor owing to aberrant visualization [2]. Accumulating data have shown that hypoxia can stimulate proliferation[3], induce angiogenesis [4], accelerate invasion [5] and is responsible for treatment resistance in HCC [6]. The adaptation of HCC cells to tissue hypoxia is of central importance for tumor progression, where inducing the ubiquitous transcription factor of hypoxia-inducible factor-1α (HIF-1α) expression appears to be a critical step [7].

HIF-1α is a master regulator of essential adaptive responses to hypoxia, whose expression and transcriptional activity increasing exponentially with decreases in levels of cellular oxygen. In tumors, HIF-1α regulates proliferation, apoptosis, metastatic spread, and glucose metabolism by acting as a transcription factor for crucial proteins [8].

Previous studies have been focusing on the clinical significance as well as experimental models of HIF-1α in many types of cancer, which have led to somewhat controversial results [9,10]. On the basis of its unquestioned role as a central regulator of tumor pathophysiology, elucidating HIF-1α's prognostic value in HCC is of great clinical importance, which may lead to better patient stratification and provide rational for hypoxia targeted therapies.

Importantly, the presence of hypoxia is always associated with and accompanied by inflammation and angiogenesis [11,12]. Specifically, HIF-1α and the oncogene MYC, which is also a transcription factor, act in concert to "fine tune" cancer cells' adaptive responses to hypoxic environments [13].

In the present study, we measured mRNA expression of HIF-1α in tumor tissue from 110 randomly selected HCC patients. We found that HIF-1α expression was associated with aggressive phenotypes of HCC and could be an independent prognostic factor for HCC patients after curative hepatectomy. Meanwhile, we presented the real-time reverse-transcriptase polymerise-chain-reaction (RT-PCR) study in the same cohort of patients on a battery of core genes which play important roles in inflammation (COX-2, MMP7, MMP9) [14-16] and angiogenesis (VEGF, PDGFRA) [17,18], as well as MYC which was confirmed as a key hypoxia regulator [19], to find the correlation of HIF-1α with these factors that can also affect and reflect tumor behaviors. These factors were selected for at least two of the three aspects: being crucial in inflammation or angiogenesis; being active in tumor metastasis or invasion; being mechanistically related to hypoxia. At last, using in situ immunostaining of HIF-1α protein, we got a further confirmation of the clinical significance of this crucial factor.

## Methods

### Patients and Specimens

Under the following the inclusion and exclusion criteria: (a) distinctive pathologic diagnosis of HCC, (b) without anticancer treatment and distant metastases before surgery, (c) underwent primary and curative resection for HCC between 2002 and 2005, defined as macroscopically complete removal of the tumor, as described previously [20], and (d) with complete clinicopathologic and follow-up data, about 2000 patients underwent hepatectomy at Zhongshan Hospital, Fudan University between 2002 and 2005 were identified. Following approval by the Institutional Review Board, a total of 110 patients in this time, with available frozen tumor specimens from our prospectively established tissue bank, were chosen randomly to be entered in this retrospective study. The mean age of patients was 52.4 years (range: 28-75) and all the patients were classified with Barcelona Clinic Liver Cancer staging system (BCLC stage) [21]. Informed consent was obtained from all patients.

A diagnosis of recurrence was confirmed by an elevated AFP level and typical imaging appearance in computed tomography and/or magnetic resonance imaging scan [20]. Overall survival (OS) was defined as the interval from surgery and death. Disease-free survival time (DFS) was defined as the interval from surgery to recurrence. The data was censored on last follow-up for living patients or for the patients in whom tumor recurrence was not diagnosed. The median follow-up time was 24.0 months (range: 1.5-68.0 mon; SE: 1.49).

### Real-time RT-PCR

Total RNA was isolated from each specimen with Trizol reagent (Invitrogen, Carlsbad, CA) and then reverse transcribed with the oligo dT primers and SuperScript RT (Invitrogen). The PCR primers used were showed in Table 1. Quantitative real-time RT-PCR was performed by using a 384-well ABI 7900 HT (Applied Biosystems, Foster City, CA). In particular, for a single gene, PCR amplifications of all the 110 specimens were simultaneously performed on the same 384-well plate in duplicate to avoid potential variations. Duplicate RT-PCR samples in each assay were collapsed by averaging.

**Table 1:** Forward and reverse primers for the genes analyzed

Gene	Forward primer	Reverse primer
HIF-1α	5'CTGCTGTCTTACTGGTCCTT3'	5'GTCGCTTCTCCA ATTCTTAC3'
COX-2	5'CCATTCAGTTCCCACCATCT3'	5'TCACTGCTGTTGGGTCTCTG3'
MMP7	5'AGATGTGGAGTGCCAGATGT3'	5'TAGACTGCTACCATCCGTCC3'
MMP9	5'GGCGCTCATGTACCCTATGT3'	5'CCTGTGTACACCCACACCTG3'
VEGF	5'ATGAACTTTCTGCTCTCTGG3'	5'TCATCTCTCCTATGTGCTGGC3'
PDGFRA	5'GGGGAAACGATTGTGGTCACC3'	5'CCCGCACCTCTACAACAAAAT3'
MYC	5'AAAGGCCCCCAAGGTAGTTA3'	5'TTTCCGCAACAAGTCCTCTT3'
HPRT	5'CCTGGCGTCGTGATTAGTG3'	5'CAGAGGGCTACAATGTGATGG3'

TBP	5'ACCACTCCACTGTATCCCTCC3'	5'CTGTTCTTCACTCTTGGCTCCT3'

Cycling parameters were as follows: 2 min at 50°C and 10 min at 95°C followed by 40 cycles of 30 sec at 95°C and 2 min at 60°C. The relative changes in gene expression were calculated by the ΔΔCt method using the SDS 2.1 software according to the manufacturer's instructions (Applied-Biosystems). The expression of each gene was related to its expression in the reference RNA pool from the 10 normal liver tissues used as a calibrator. To control for variability in cDNA quantity, integrity, and individual primer efficiency, data were normalized against two housekeeping genes (TBP and HPRT) as we previously described [22]. (See Additional file 1: Figure S1 for relative mRNA expression of HIF-1α, COX-2, MMP7, MMP9, VEGF, PDGFRA and MYC with the cut-off value determined by the X-tile software).

### Tissue microarray and Immunohistochemistry

Tissue microarrays (TMA) were constructed as described previously [23]. Triplicates of 1-mm-diameter cylinders from representative areas of tumor center, away from necrotic, hemorrhagic and major fibrotic areas, were included in each case, along with different controls (spleen, lymph node, artery and glioma), to ensure reproducibility and homogenous staining of the slides. Serial sections (4 μm thick) were placed on slides coated with 3-aminopropyltriethoxysilane.

Immunohistochemical staining by the streptavidin-biotin-peroxidase complex method was performed as previously described. Rabbit polyclonal antibody to HIF-1α (ab65979; Abcam, Cambridge, UK) was used at a dilution of 1:50. Briefly, sections were dewaxed, hydrated, and washed. After neutralization of endogenous peroxidase and microwave antigen retrieval, slides were preincubated with blocking serum and then incubated overnight with the primary Ab. Then, the sections were serially rinsed, incubated with second antibodies, and treated with horseradish peroxidase-conjugated streptavidin. Reaction products were visualized with 3, 3'-diaminobenzidine tetrahydrochloride and counterstained with hematoxylin. For the negative control, the primary antibodies were replaced with phosphate buffered saline.

The manual evaluation of HIF-1α expression involved location and degree of reactivity. Location of expression included not only the cell type but also that of the subcellular localization (i.e., nucleus, cytoplasm). Degree of expression was determined by assessing semiquantitatively percentage of positive nuclear staining cells in random four fields of each core as well as evaluating for cytoplasmic staining intensity in the entire disk. The final result was classified as follows: I, no staining; II, nuclear staining in less than 10% of cells and/or with weak cytoplasmic staining; III, nuclear staining in 10%-50% of cells and/or with moderate cytoplasmic staining; IV, nuclear staining in more than 50% of cells and/or with strong cytoplasmic staining [24] In statistical analysis, the grades I and II were considered to be low expression of HIF-1α protein, with III and IV as high expression [25]. (See Additional file 2: Table S1 for degree of HIF-1α immunohistochemical staining)

Two independent investigators blind to the clinicopathologic data assessed the immunohistochemistry. When independent scoring of a case differed, the case was rechecked, and the final score was determined by recounting HIF-1α positive nuclear staining cells using a multiheaded microscope with both reviewers simultaneously viewing the slides.

### Statistical analysis

The statistical analysis was done with SPSS 15.0 program. To assess the relationship between experimental results and clinicopathologic characteristics, Fisher's exact test and Spearman's ρ coefficients test were carried out as appropriate. To find out the correlations between HIF-1α and other evaluated factors, Pearson Correlation test was used.

Patient survival distribution was calculated using the Kaplan-Meier method and compared with the log-rank test. Cut-point value of experiment results was determined by the X-tile software [26]. Univariate and multivariate analyses were based on the Cox proportional hazards regression model. All variables that were found to be statistical significant in univariate analysis were included in multivariate analysis. *P *< 0.05 (two-sided) was judged statistically significant.

## Results

### Correlation of HIF-1α mRNA to clinicopathologic features

According to the optimal cut-point value of HIF-1α determined by the X-tile software, the expression of HIF-1α mRNA was defined as high expression in 42 specimens (38.2%) and low expression in 68 specimens (61.8%). The HIF-1α mRNA expression level has no correlation to gender, tumor size, encapsulation, number, hepatitis history, liver cirrhosis and preoperative AFP level, while older patients seems to have tumor with higher HIF-1α expression (*P *= 0.049) (Table 2).

**Table 2:** Correlations of HIF-1α mRNA and protein with clinicopathological characters

	HIF-1α mRNA			HIF-1α protein		
	
	low	high	*P*	low	high	*P*
Patients	68	42		71	39	
Age(years)						
≤52	28	26	0.049	32	22	0.320
>52	40	16		39	17	
Sex						
Male	57	38	0.40	57	38	0.017
Female	11	4		14	1	
Hepatitis history						
Yes	61	39	0.739	62	38	0.094
No	7	3		9	1	
Preoperative AFP(ng/ml)						
≤20	29	16	0.693	33	12	0.156
>20	39	26		38	27	
Liver cirrhosis						
Yes	59	34	0.428	56	37	0.029
No	9	8		15	2	
ALT(U/L)						
≤40	36	22	1.000	38	20	0.844
>40	32	20		33	19	
Γ-GT(U/L)						
≤54	30	14	0.318	31	13	0.316
>54	38	28		40	26	
Tumor size (cm)						
≤5	35	21	1.000	33	23	0.236
>5	33	21		38	16	
Tumor encapsulation						
Yes	31	18	0.845	32	17	1.000
No	37	24		39	22	
Tumor number						
Single	53	32	0.820	56	29	0.638
Multiple	15	10		15	10	
Vascular invasion						
Yes	26	25	0.033	27	24	0.027
No	42	17		44	15	
BCLC stage						
A	30	10	0.041	31	9	0.039
B + C	38	32		40	30	
Tumor differentiation						
I+II	40	18	0.119	36	22	0.690
III+IV	28	24		35	17	
Prophylactic treatment						
Yes	33	22	0.845	31	24	0.110
No	35	20		39	16	

Detailed legends: Fisher's exact tests were used for all the analysis of correlations.

The HIF-1α mRNA expression level showed significantly positive correlation with vascular invasion. In all the 110 patients, 59 cases without tumor vascular invasion have a high expression rate of 28.8%, as compared with the high expression rate of 49.0% (*P *= 0.033) in cases with vascular invasion. Also, the HIF-1α mRNA expression level was significantly correlated to BCLC stages. The HIF-1α mRNA expression in patients at stage A was lower than those at stages B and C (*P *= 0.041) (Table 2).

### Correlation of HIF-1α to COX-2, MMP7, MMP9, VEGF, PDGFRA and MYC

Significant positive correlations were observed between HIF-1α mRNA and the putative markers of inflammation, angiogenesis and MYC: COX-2 (*P *< 0.001, *r *= 0.708), MMP7 (*P *< 0.001, *r *= 0.593), MMP9 (*P *< 0.001, *r *= 0.384), PDGFRA (*P *< 0.001, *r *= 0.493) and MYC (*P *< 0.001, *r *= 0.230). However, there were no significant correlation between mRNA expression level of HIF-1α and VEGF (*P *< 0.183, *r *= 0.128). Of all the six parameters, COX-2, MMP7 and PDGFRA were the top three factors with the largest correlation coefficients to HIF-1α (Table 3).

**Table 3:** Correlations between HIF-1α mRNA and other molecular markers as well as prognostic significance.

	HIF-1α	COX-2	MMP7	PDGFRA	MMP9	MYC	VEGF
Correlation (*P*)	0.000	< 0.001	< 0.001	< 0.001	< 0.001	0.016	0.183
Coefficient (*r*)	1.000	0.708	0.593	0.493	0.384	0.230	0.128
OS (*P*)	0.012	0.004	0.010	0.010	NS	NS	NS
DFS (*P*)	0.004	0.010	NS	0.038	NS	NS	NS

Detailed legends: The *P *values for correlation were determined by spearman's coefficience. The *P *values for OS and DFS were calculated using multivariate Cox proportional hazards analysis. All factors were presented in different multivariable analysis respectively and variables were adopted for their prognostic significance by univariate analysis. NS means not significant (*P *> 0.05).

### Prognosis

The OS and DFS rates were 80.0% and 67.6% at 1 year, and 51.4% and 43.7% at 5 years respectively, for the whole study population.

On univariate analysis, sex, hepatitis history, cirrhosis, ALT level showed no prognostic significance for OS and DFS. AFP, γ-GT, tumor differentiation, size, vascular invasion and encapsulation were found to be significant predictors for OS. Age, AFP, γ-GT, tumor size, number, vascular invasion and encapsulation were predictors for DFS (Table 4).

**Table 4:** Univariate analyses of factors associated with survival and recurrence

Variables	OS		DFS	
	
	Hazard ratio(95%CI)	*P*	Hazard ratio(95%CI)	*P*
Age (year)	0.982 (0.958-1.006)	0.142	0.976 (0.953-0.999)	0.043
Sex (female vs. male)	0.630 (0.250-1.588)	0.298	0.673 (0.288-1.574)	0.361
Hepatitis history (no vs. yes)	1.384 (0.498-3.848)	0.534	0.874 (0.395-1.935)	0.739
Liver cirrhosis (no vs. yes)	1.855 (0.746-4.657)	0.190	0.904 (0.455-1.797)	0.773
AFP(ng/ml) (≤ 20 vs. >20)	2.183 (1.175-4.035)	0.013	2.553 (1.401-4.651)	0.002
ALT(U/L) (≤ 40 vs. >40)	0.877 (0.502-1.533)	0.645	0.817 (0.476-1.401)	0.463
γ-GT(U/L) (≤ 54 vs. >54)	2.475 (1.313-4.664)	0.005	1.555 (0.888-2.722)	0.123
Tumor differentiation (I+II vs. III+IV)	1.860 (1.062-3.297)	0.030	1.180 (0.691-2.014)	0.545
Tumor size (cm)	1.179 (1.109-1.253)	0.001	1.152 (1.083-1.226)	0.001
Tumor number (single vs. multiple)	1.597 (0.860-2.966)	0.138	2.851 (1.611-5.046)	0.001
Vascular invasion (no vs. yes)	5.706 (2.971-10.960)	0.001	4.028 (2.288-7.089)	0.001
Encapsulation (complete vs. no)	2.060 (1.135-3.737)	0.017	3.273 (1.773-6.044)	0.001
BCLC stage (A vs. B+C)	6.225 (2.645-14.647)	0.001	2.601 (1.390-4.867)	0.003
COX-2 mRNA (low vs. high)	2.042 (1.157-3.604)	0.014	1.839 (1.068-3.167)	0.027
MMP7 mRNA (low vs. high)	2.278 (1.297-4.001)	0.004	0.753 (0.409-1.384)	0.060
MMP9 mRNA (low vs. high)	0.740 (0.412-1.328)	0.313	1.711 (0.977-2.995)	0.609
VEGF mRNA (low vs. high)	1.370 (0.756-2.483)	0.299	1.906 (1.096-3.126)	0.022
PDGFRA mRNA (low vs. high)	1.801 (1.017-3.188)	0.044	1.771 (1.023-3.066)	0.041
MYC mRNA (low vs. high)	0.948 (0.517-1.735)	0.862	0.867 (0.501-1.499)	0.361
HIF-1α mRNA (low vs. high)	2.644 (1.524-4.655)	0.001	2.514 (1.462-4.324)	0.001
HIF-1α protein (low vs. high)	1.874 (1.074-3.270)	0.027	2.004 (1.167-3.440)	0.012

Detailed legends: The *P *values were calculated using univariate Cox proportional hazards analysis. Cut-point values of the experiment results were determined by the X-tile software.

**Figure 1:**
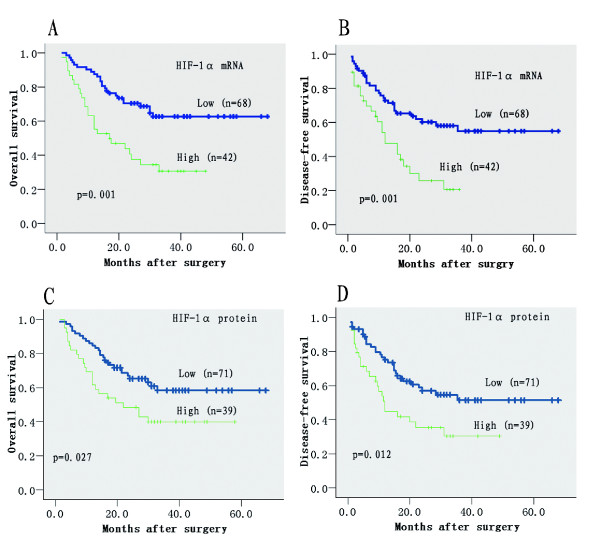
**Kaplan-Meier analysis of overall and disease-free survival for HIF-1α mRNA and protein**. Low expression of HIF-1α mRNA (A, B) or protein (C, D) was associated with both prolonged survival and reduced recurrence. The P values were determined by the log-rank test.

Univariate analysis revealed that the HIF-1α mRNA expression level was associated with both OS (P = 0.001) and DFS (P = 0.001) (Table 4; Figure 1A and 1B). The OS rate for patients with high and low expression of HIF-1α mRNA were 63.2% and 88.9% at 1 year (P = 0.001), 30.4% and 62.5% at 5 year (*P *= 0.001) respectively. Similarly, the DFS rate for patients with high and low expression were 53.9% and 74.3% at 1 year (*P *= 0.004), 20.4% and 54.8% at 5 year (*P *= 0.004) respectively.

Further, multivariate analysis confirmed that HIF-1α mRNA expression was independent prognostic factor for OS (*P *= 0.012, hazard ratio: 2.167, 95%CI: 1.185-3.965) and DFS (*P *= 0.004, hazard ratio: 2.359, 95%CI: 1.325-4.201). In addition, for DFS, the *P *value was the smallest and the hazard ratio was the largest among these factors (Table 5 and Additional files 3, 4, 5, 6, 7 and 8 ).

**Table 5:** Multivariate analyses of factors associated with survival and recurrence

	Hazard ratio (95%CI)	*P*
OS		
AFP(ng/ml) (≤ 20 vs. >20)	1.320 (0.666-2.615)	0.426
γ-GT(U/I) (≤ 54 vs. >54)	2.098 (1.073-4.103)	0.030
Tumor differentiation (I+II vs. III+IV)	1.228 (0.661-2.279)	0.516
Tumor size (cm)	1.102 (1.016-1.196)	0.020
Vascular invasion (no vs. yes)	4.351 (1.992-9.500)	0.001
Capsule (complete vs. no)	0.676 (0.332-1.375)	0.280
HIF-1α mRNA (low vs. high)	2.167 (1.185-3.965)	0.012
COX-2 mRNA (low vs. high)	2.557 (1.355-4.824)	0.004
MMP7 mRNA (low vs. high)	2.287 (1.217-4.301)	0.010
PDGFRA mRNA (low vs. high)	2.320 (1.225-4.394)	0.010
HIF-1α protein (low vs. high)	2.108 (1.120-3.969)	0.021
DFS		
Age (year)	0.990 (0.963-1.018)	0.475
AFP(ng/ml) (≤ 20 vs. >20)	1.707 (0.903-3.227)	0.100
Tumor size (cm)	1.090 (0.997-1.191)	0.060
Tumor number (single vs. multiple)	3.324 (1.818-6.079)	0.001
Vascular invasion (no vs. yes)	2.453 (1.192-5.047)	0.015
Encapsulation (complete vs. no)	1.573 (0.781-3.168)	0.204
HIF-1α mRNA (low vs. high)	2.359 (1.325-4.201)	0.004
COX-2 mRNA (low vs. high)	2.170 (1.201-3.921)	0.010
MMP7 mRNA (low vs. high)	1.499 (0.830-2.707)	0.179
VEGF mRNA (low vs. high)	1.702 (0.936-3.094)	0.081
PDGFRA mRNA (low vs. high)	1.910 (1.038-3.514)	0.038
HIF-1α protein (low vs. high)	2.265 (1.251-4.009)	0.007

Detailed legends: The *P *values were calculated using multivariate Cox proportional hazards analysis. Variables were adopted for their prognostic significance by univariate analysis and no significant correlation between each other. COX-2 mRNA, PDGFRA mRNA, MMP7 mRNA, VEGF mRNA and HIF-1α protein were presented in different multivariable analysis respectively. (For details see Additional files 3, 4, 5, 6, 7 and 8)

Also, as for mRNA expression, COX-2 and PDGFRA, being correlated with HIF-1α with larger coefficients among the six parameters, showed significance for both OS and DFS on univariate analysis (COX-2: *P *= 0.014 for OS, *P *= 0.027 for DFS; PDGFRA: *P *= 0.044 for OS, *P *= 0.041 for DFS) and then strengthened on multivariate analysis (COX-2: *P *= 0.004 for OS, *P *= 0.010 for DFS; PDGFRA: *P *= 0.010 for OS, *P *= 0.038 for DFS). MMP7, which also correlated with HIF-1α, showed significance for OS on both uni- and multi-variate analyses, and patients with high MMP7 had a propensity of increased recurrence (*P *= 0.060). However, VEGF was only significant for DFS (*P *= 0.022) but has no influence on OS (*P *= 0.299) in univariate analysis. On multivariate analysis, VEGF was not a significant prognostic factor for recurrence (*P *= 0.081) any more. Neither MMP9 (*P *= 0.313 for OS, *P *= 0.607 for DFS) nor MYC (*P *= 0.862 for OS, *P *= 0.361 for DFS) had any prognostic value for either OS or DFS in HCC. (Tables 4 and 5)

### Immunohistochemical confirmation

**Figure 2:**
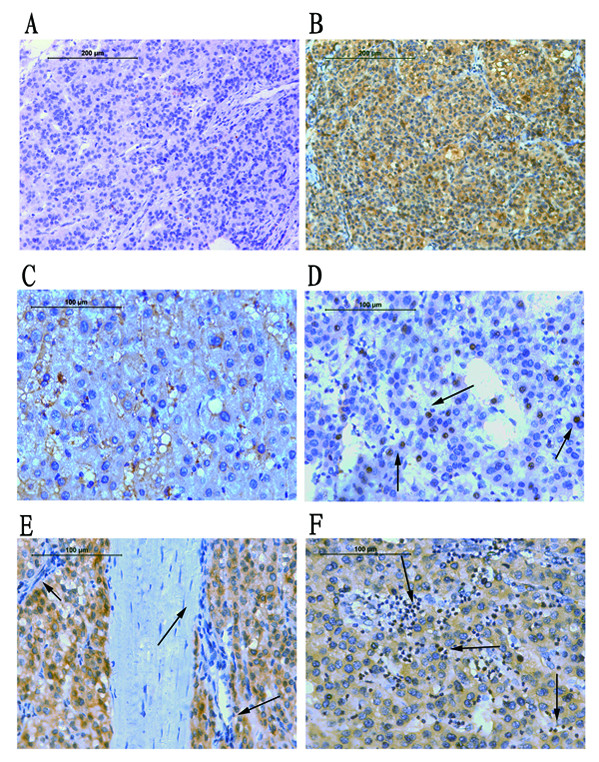
**Representative images of immunohistochemical staining**. A: Negative expression of HIF-1α protein (× 200). B: Positive expression of HIF-1α protein (× 200). C: HIF-1α protein localized in the cytoplasm (× 400). D: HIF-1α protein localized in the nucleus (arrows) (× 400). E: Negative staining in fibroblasts and endothelial cells (arrows) (× 400). F: Positive staining in lymphocytes (arrows) (× 400).

In HCC, positive staining of HIF-1α protein was observed mainly in cancer cells, and occasionally in infiltrating lymphocytes as well. In contrast, the fibroblasts and endothelial cells in tumor tissue always showed negative staining. HIF-1α protein was commonly detected in cytoplasm, whereas in some cases HIF-1α protein was detected mostly in nucleus. Representative images were showed in Figure 2.

According to the optimal cut-point determined by the X-tile software, there were 71 (64.5%) patients with low expression and 39 (35.5%) patients with high expression. Although the immunohistochemical results were not totally in parallel with the RT-PCR results, protein expression of HIF-1α were highly correlated with the mRNA expression (*P *= 0.001). (See Additional file 9: Table S2 for correlations between HIF-1α mRNA and protein)

The tumor HIF-1α protein expression level has no association with patient age, tumor size, number, encapsulation, hepatitis history and preoperative AFP. Similar to HIF-1α mRNA, the tumor HIF-1α protein expression level was significantly associated with tumor vascular invasion (*P *= 0.027) and BCLC stages (*P *= 0.039). (Table 2)

High expression of protein also means the poor outcome. Both OS and DFS were better in low expression group than that of high expression group. The OS rates for patient with high and low expression of HIF-1α protein were 69.2% and 85.9% at 1 year (*P *= 0.015), 39.8% and 58.0% at 5 years (*P *= 0.015) respectively. Also, the DFS rates for high and low expression were 50.8% and 76.5% at 1 year (*P *= 0.009), 30.3% and 51.2% at 5 year (*P *= 0.009) respectively.

On univariate analysis, tumor HIF-1α protein expression level was significant for both OS (*P *= 0.027) and DFS (*P *= 0.012) (Table 4; Figure 1C and 1D), which were further authenticated on multivariate analysis (*P *= 0.021 for OS; *P *= 0.007 for DFS) (Table 5). These may indicate that the prognostic value of HIF-1α was further validated on protein level.

## Discussion

It has been established that human solid tumors develop a pathophysiologic microenvironment during growth, characterized by an irregular microvascular network and regions of hypoxia [7]. Of clinical importance, hypoxia-related genes and proteins are potentially alternative endogenous markers, as compared with the inability and the cost of exogenous hypoxia markers [2]. In this study, we demonstrated for the first time that HIF-1α, at both mRNA and protein levels, is an independent prognostic factor for both survival and recurrence in HCC with the smallest *P *value as well as the largest hazard ratio for recurrence. In addition, we found that high expression of HIF-1α was significantly associated with an advanced stage and aggressive phenotypes. Several in vitro experimental studies sustained our results. In rat HCC models, tumor progression after hypoxia and chemotherapy was related to up-regulation of HIF-1α and subsequent VEGF production, and transcriptional blockade of HIF-1α could enhance their therapeutic efficacy [27]. In cell lines, HCC cell proliferation was inhibited by HIF-1α antisense oligonuclecotide [28]. As a key player in tumor progression, HIF-1α overexpression is associated with an increased mortality and treatment failure in various cancers although conflicting data exist [7]. The current study emphasizes the clinical significance of HIF-1α, suggesting that related therapy in the future is promising in reducing recurrence and prolonging survival [29].

The underlying mechanisms that HIF-1α affects metastatic spread and selecting cells with more aggressive phenotypes have not been fully defined, but recent studies have shown a role for increased invasive capacity and inflammation [11]. Consistently, we found the importance of HIF-1α in HCC is associated with or may contribute to other markers related to inflammation, angiogenesis and invasion. COX-2, MMP7 and MMP9 were well-recognized factors in inflammation conditions [14-16]. Among these molecules, COX-2, being involved in the chronic inflammation-related development of HCC, correlated with HIF-1α with the largest correlation coefficient (*r *= 0.708). Mechanistically, hypoxia potently triggers COX-2 transcription in many kinds of cells in primary culture [30,31]. Also, hypoxia influences the expression of MMPs although the mechanisms are controversial [5,32]. The mRNA expression of MMP7, which can degrade various ECM proteins and support the role in tumor invasion and spread in HCC, has significant correlation to HIF-1α mRNA expression in our study (*r *= 0.593). However, MMP9, without any prognostic signifcance, seems to have less significant correlation to HIF-1α mRNA than MMP7 in HCC (*r *= 0.383). VEGF and PDGFRA promote the tumor angiogenesis in inflammation and hypoxia conditions [18,33]. HIF-1α was often considered a master regulator of VEGF expression and angiogenesis in hypoxia. However, in our study, VEGF has no significant correlations with HIF-1α. As suggested, the regulation of VEGF expression may be come true by HIF-1α-independent pathways in hypoxia [34]. Otherwise, studies have revealed hypoxia can upregulate the expression of PDGFA [10], PDGFB [35] and PDGFRB [36], with little information on the correlation between HIF-1α and PDGFRA. Our study provide the preliminary data that HIF-1α positively correlated with and thus may upregulate PDGFRA, also a crucial angiogenic factor, in HCC (*P *< 0.001, *r *= 0.493).

All of these factors were conceived to promote tumor progression. Some of these molecules, such as COX-2, PDGFRA and MMP7, were believed to be prognostic factors in HCC according to our research. COX-2 may regulate HCC growth by COX-2-derived PG signaling pathway [37]. PDGFRA may regulate tumor angiogenesis by PDGFRA-p70S6K pathway which is related to the function of fibroblast growth factor (FGF) and expression of VEGF and hepatocytes growth factor (HGF) [18]. MMPs increase HCC invasion and growth through the degradation of extracellar matrix [38].

However, we propose that HIF-1α, with the smallest *P *value as well as the largest hazard ratio for DFS among these factors, may be the central factor to affect HCC outcome. Extensive researches have confirmed that HIF-1α controls many hundreds of target genes which play important roles in the cellular adaptation to hypoxia, and the proteins of these genes are involved in processes which can make cancer cells much more aggressive, such as angiogenesis, proliferation, apoptosis, energy metabolism and glucose transportation [29].

In addition, the oncogene MYC also plays a crucial role in hypoxic response, and cooperates with HIF-1α to alter cellular metabolism and promote cancer progression [19]. In our study, MYC was positive correlated with HIF-1α (*P *= 0.016). However, the correlation coefficient was small (*r *= 0.230), and MYC has no prognostic value in HCC. The findings that MYC expression was repressed in the human hepatoma cell lines under extremely low oxygen concentrations may partly responsible for this non-significance [39].

In summary, HIF-1α was an independent prognosticator for both survival and recurrence in HCC. Although the correlations between HIF-1α and markers of inflammation, angiogenesis and the cooperative MYC oncogene are not very clear, we can hypothesize that HIF-1α was the crucial factor in HCC progression on the basis of our findings. The therapy targeting HIF-1α and associated molecules may profoundly reduce recurrence and prolong survival.

## Conclusion

HIF-1α was an independent prognosticator for both survival and recurrence in HCC. Markers of inflammation, angiogenesis and the cooperative MYC oncogene, who have closer correlation with HIF-1α, seem to be better for prognostic stratification, suggesting that HIF-1α was one of the most crucial factors in HCC progression. Attaching importance to HIF-1α and related molecules may improve the prognostic stratification and therapeutic technique.

## Abbreviations

AFP: alpha-fetoprotein; BCLC stage: Barcelona Clinic Liver Cancer stage; CI: confidence interval; COX-2: cyclooxygenase-2; DFS: disease-free survival; HBV: hepatitis B virus; HCC: hepatocellular carcinoma; HIF-1α: hypoxia-inducible factor 1, alpha subunit; HPRT: hypoxanthine-guanine phosphoribosyltransferase; MMP: matrix metalloproteinase; OS: overall survival; PDGFRA: platelet-derived growth factor receptor, alpha polypeptide; RT-PCR: polymerase chain reaction; TBP: TATA binding protein; VEGF: vascular endothelial growth factor.

## Competing interests

The authors declare that they have no competing interests.

## Authors' contributions

JF developed the study concept, aims and initiated the project. QG and CXD performed the experimental work described in the study and were both responsible for the drafting of the manuscript. SJQ supervised the process of the whole research. JZ and BHZ provided valuable scientific suggestions. MJJ, MYC and YFX have participated in its design and coordination. All authors have read and approved the final manuscript.

## Pre-publication history

The pre-publication history for this paper can be accessed here:

http://www.biomedcentral.com/1471-2407/9/418/prepub

## Supplementary Material

Additional file 1 **Figure S1: Relative expression of HIF-1α, COX-2, MMP7, MMP9, VEGF, PDGFRA and MYC with the cut-off value determined by the X-tile software**. The blue dots show the relative value of mRNA expression. The red lines show the cut-off value. Click here for file

Additional file 2 **Table S1: Degree of HIF-1α immunohistochemistry in tissue array of HCC**. I, no staining; II, nuclear staining in less than 10% of cells and/or with weak cytoplasmic staining; III, nuclear staining in 10%-50% of cells and/or with moderate cytoplasmic staining; IV, nuclear staining in more than 50% of cells and/or with strong cytoplasmic staining. Click here for file

Additional file 3 Figure S2: Kaplan-Meier analysis of OS and DFS for COX-2, MMP7, MMP9, VEGF, PDGFRA and MYC mRNA Click here for file

Additional file 4 Table S3: Multivariate analyses of variables associated with survival and recurrence including mRNA expression of COX-2 as co-variable Click here for file

Additional file 5 Table S4: Multivariate analyses of variables associated with survival and recurrence including mRNA expression of PDGFRA as co-variable Click here for file

Additional file 6 Table S5: Multivariate analyses of variables associated with survival and recurrence including mRNA expression of MMP7 as co-variable Click here for file

Additional file 7 **Table S6: Multivariate analyses of variables associated with survival and recurrence including protein expression of HIF-1α as co-variable**. (P = 0.021 for OS, P = 0.007 for DFS). Click here for file

Additional file 8 **Table S7: Multivariate analyses of variables associated with survival and recurrence including mRNA expression of VEGF as co-variable**. (P = 0.081 for DFS). Click here for file

Additional file 9 Table S2: Correlations between HIF-1α mRNA and protein expression. Click here for file
