# Supplementary material for: Hypoxia-inducible factor-1 alpha, in association with inflammation, angiogenesis and MYC, is a critical prognostic factor in patients with HCC after surgery
Source: BMC Cancer. 2009 Dec 1;9:418. doi: 10.1186/1471-2407-9-418 (PMC2797816; doi:10.1186/1471-2407-9-418)
Supplement: Additional file 1 — Figure S1: Relative expression of HIF-1α, COX-2, MMP7, MMP9, VEGF, PDGFRA and MYC with the cut-off value determined by the X-tile software. The blue dots show the relative value of mRNA expression. The red lines show the cut-off value. [file 1471-2407-9-418-S1.DOC]

Figure S1: **Relative expression of HIF-1α, COX-2, MMP7, MMP9, VEGF, PDGFRA and MYC with the cut-off value determined by the X-tile software.**


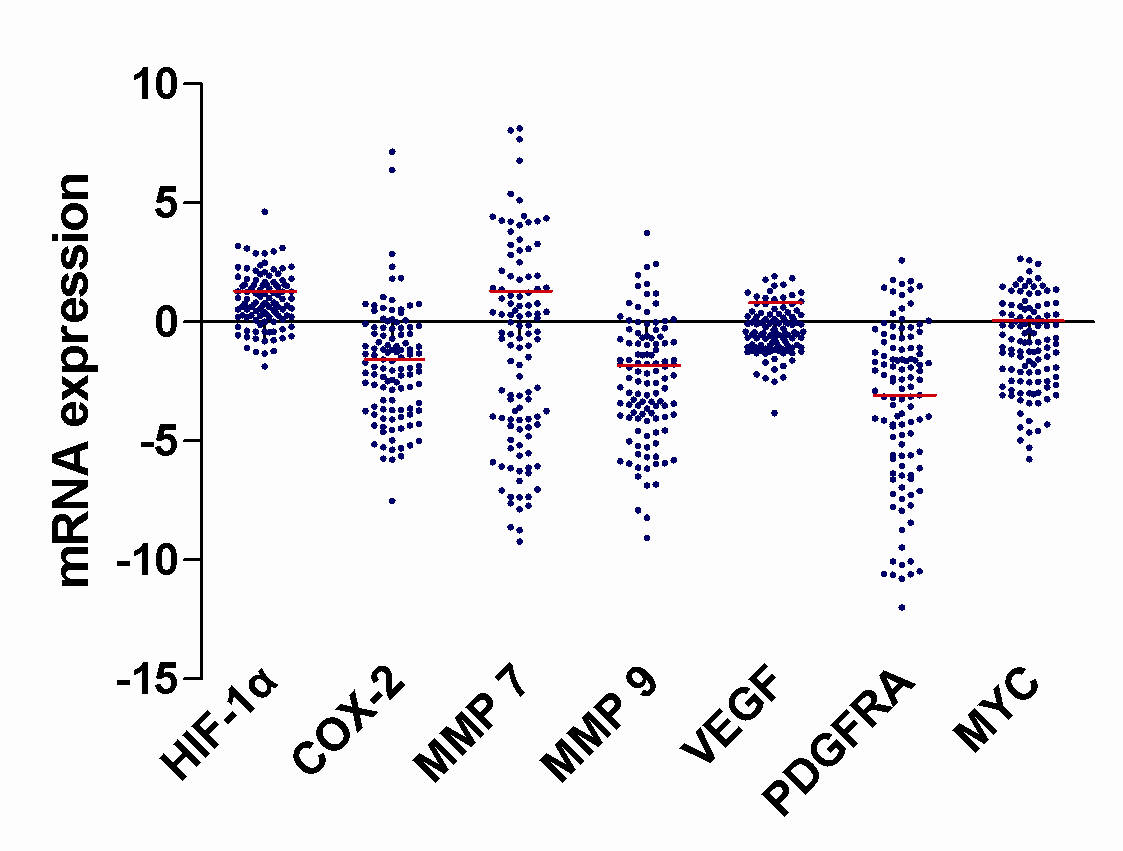


The blue dots show the relative value of mRNA expression. The red lines show the cut-off value.
