# Supplementary material for: Hypoxia-inducible factor-1 alpha, in association with inflammation, angiogenesis and MYC, is a critical prognostic factor in patients with HCC after surgery
Source: BMC Cancer. 2009 Dec 1;9:418. doi: 10.1186/1471-2407-9-418 (PMC2797816; doi:10.1186/1471-2407-9-418)
Supplement: Additional file 2 — Table S1: Degree of HIF-1α immunohistochemistry in tissue array of HCC. I, no staining; II, nuclear staining in less than 10% of cells and/or with weak cytoplasmic staining; III, nuclear staining in 10%-50% of cells and/or with moderate cytoplasmic staining; IV, nuclear staining in more than 50% of cells and/or with strong cytoplasmic staining. [file 1471-2407-9-418-S2.DOC]

Table S1: **Degree of HIF-1α immunohistochemical staining in tissue array of HCC**

| staining degree | I | II | III | IV |
| --- | --- | --- | --- | --- |
| Number of specimens | 21 | 50 | 32 | 7 |
| percentage | 19.1% | 45.5% | 29.1% | 6.4% |

I, no staining; II, nuclear staining in less than 10% of cells and/or with weak cytoplasmic staining; III, nuclear staining in 10%-50% of cells and/or with moderate cytoplasmic staining; IV, nuclear staining in more than 50% of cells and/or with strong cytoplasmic staining
