# Supplementary material for: Hypoxia-inducible factor-1 alpha, in association with inflammation, angiogenesis and MYC, is a critical prognostic factor in patients with HCC after surgery
Source: BMC Cancer. 2009 Dec 1;9:418. doi: 10.1186/1471-2407-9-418 (PMC2797816; doi:10.1186/1471-2407-9-418)

Figure S2: **Kaplan-Meier analysis of OS and DFS for COX-2, MMP7, MMP9, VEGF, PDGFRA and MYC mRNA.** COX-2 (P=0.014), MMP7 (P=0.004), PDGFRA (P=0.044) had prognostic significance for OS, while COX-2 (P=0.003), VEGF(P=0.022), PDGFRA (P=0.041) had prognostic significance for DFS.


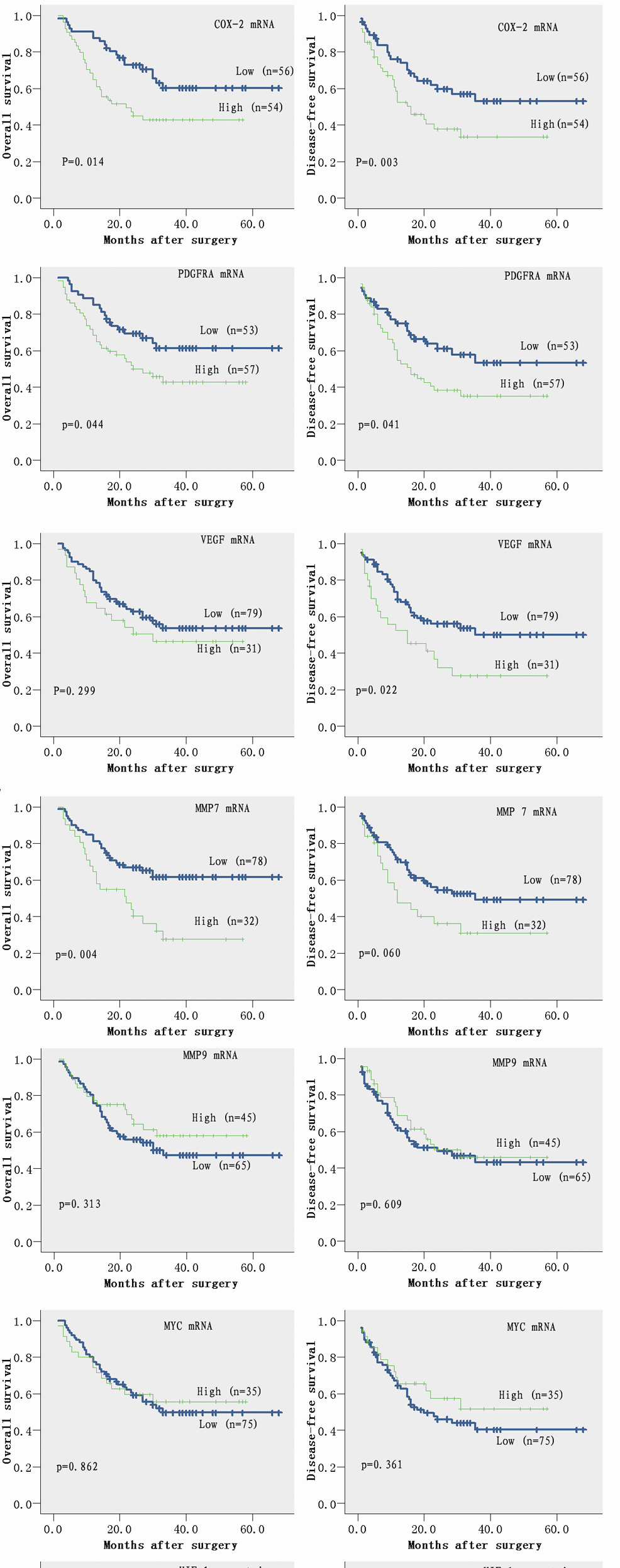

Supplement: Additional file 3 — Figure S2: Kaplan-Meier analysis of OS and DFS for COX-2, MMP7, MMP9, VEGF, PDGFRA and MYC mRNA [file 1471-2407-9-418-S3.DOC]
