# Supplementary material for: Hypoxia-inducible factor-1 alpha, in association with inflammation, angiogenesis and MYC, is a critical prognostic factor in patients with HCC after surgery
Source: BMC Cancer. 2009 Dec 1;9:418. doi: 10.1186/1471-2407-9-418 (PMC2797816; doi:10.1186/1471-2407-9-418)
Supplement: Additional file 4 — Table S3: Multivariate analyses of variables associated with survival and recurrence including mRNA expression of COX-2 as co-variable [file 1471-2407-9-418-S4.DOC]

Table S2: **Multivariate analyses of** **variables associated with survival and recurrence including mRNA expression of COX-2 as co-variable.**

|  | Hazard ratio (95%CI) | *P* |
| --- | --- | --- |
| OS |  |  |
| AFP(ng/ml) (≤20 vs.＞20) | 1.186 (0.601-2.341) | 0.624 |
| γ-GT(U/L) (≤54 vs. ＞54) | 1.804 (0.908-3.581) | 0.092 |
| Tumor differentiation (Ⅰ+Ⅱ vs.Ⅲ+Ⅳ) | 1.386 (0.764-2.515) | 0.282 |
| Tumor size（cm） | 1.132 (1.038-1.235) | 0.005 |
| Vascular invasion (no vs. yes) | 4.743 (2.178-10.333) | <0.001 |
| Encapsulation (complete vs. no) | 0.667 (0.327-1.361) | 0.266 |
| COX-2 mRNA (low vs. high) | 2.557 (1.355-4.824) | 0.004 |
| DFS |  |  |
| Age (year) | 1.060 (0.589-1.909) | 0.845 |
| AFP(ng/ml) (≤20 vs.＞20) | 1.657 (0.875-3.136) | 0.121 |
| Tumor size（cm） | 1.099 (1.006-1.200) | 0.037 |
| Tumor number (single vs. multiple) | 2.950 (1.632-5.335) | <0.001 |
| Vascular invasion (no vs. yes) | 2.485 (1.230-5.021) | 0.011 |
| Encapsulation (complete vs. no) | 1.659 (0.809-3.402) | 0.167 |
| COX-2 mRNA (low vs. high) | 2.170 (1.201-3.921) | 0.010 |

Multivariate analysis, Cox proportional hazards regression model

Variables were adopted for their prognostic significance by univariate analysis and no significant correlation between each other.
