# Supplementary material for: Hypoxia-inducible factor-1 alpha, in association with inflammation, angiogenesis and MYC, is a critical prognostic factor in patients with HCC after surgery
Source: BMC Cancer. 2009 Dec 1;9:418. doi: 10.1186/1471-2407-9-418 (PMC2797816; doi:10.1186/1471-2407-9-418)
Supplement: Additional file 5 — Table S4: Multivariate analyses of variables associated with survival and recurrence including mRNA expression of PDGFRA as co-variable [file 1471-2407-9-418-S5.DOC]

Table S3: **Multivariate analyses of variables associated with survival and recurrence including mRNA expression of PDGFRA as co-variable**

|  | Hazard ratio (95%CI) | *P* |
| --- | --- | --- |
| OS |  |  |
| AFP(ng/ml) (≤20 vs.＞20) | 1.127 (0.564-2.249) | 0.735 |
| γ-GT(U/I) (≤54 vs. ＞54) | 1.875 (1.930-3.710) | 0.080 |
| Tumor differentiation (Ⅰ+Ⅱ vs.Ⅲ+Ⅳ) | 1.451 (0.795-2.648) | 0.225 |
| Tumor size（cm） | 1.133 (1.032-1.244) | 0.009 |
| Vascular invasion (no vs. yes) | 4.761 (2.179-10.402) | <0.001 |
| Encapsulation (complete vs. no) | 0.608 (0.300-1.232) | 0.168 |
| PDGFRA mRNA (low vs. high) | 2.320 (1.225-4.394) | 0.010 |
| DFS |  |  |
| Age (year) | 1.022 (0.571-1.831) | 0.940 |
| AFP(ng/ml) (≤20 vs.＞20) | 1.613 (0.845-3.082) | 0.148 |
| Tumor size（cm） | 1.105 (1.009-1.209) | 0.031 |
| Tumor number (single vs. multiple) | 2.849 (1.578-5.142) | 0.001 |
| Vascular invasion (no vs. yes) | 2.464 (1.216-4.991) | 0.012 |
| Encapsulation (complete vs. no) | 1.583 (0.772-3.249) | 0.210 |
| PDGFRA mRNA (low vs. high) | 1.910 (1.038-3.514) | 0.038 |

Multivariate analysis, Cox proportional hazards regression model

Variables were adopted for their prognostic significance by univariate analysis and no

obvious correlation between each other.
