# Supplementary material for: Hypoxia-inducible factor-1 alpha, in association with inflammation, angiogenesis and MYC, is a critical prognostic factor in patients with HCC after surgery
Source: BMC Cancer. 2009 Dec 1;9:418. doi: 10.1186/1471-2407-9-418 (PMC2797816; doi:10.1186/1471-2407-9-418)
Supplement: Additional file 6 — Table S5: Multivariate analyses of variables associated with survival and recurrence including mRNA expression of MMP7 as co-variable [file 1471-2407-9-418-S6.DOC]

Table S4: **Multivariate analyses of variables associated with survival and recurrence including mRNA expression of MMP7 as co-variable**

|  | Hazard ratio (95%CI) | *P* |
| --- | --- | --- |
| OS |  |  |
| AFP(ng/ml) (≤20 vs.＞20) | 1.229 (0.624-2.424) | 0.551 |
| γ-GT(U/I) (≤54 vs. ＞54) | 1.749 (0.868-3.522) | 0.118 |
| Tumor differentiation (Ⅰ+Ⅱ vs.Ⅲ+Ⅳ) | 1.455 (0.797-2.656) | 0.222 |
| Tumor size（cm） | 1.113 (1.020-1.215) | 0.016 |
| Vascular invasion (no vs. yes) | 5.303 (2.392-11.759) | <0.001 |
| Encapsulation (complete vs. no) | 0.529 (0.247-1.135) | 0.102 |
| MMP7 mRNA (low vs. high) | 2.287 (1.217-4.301) | 0.010 |
| DFS |  |  |
| Age (year) | 0.932 (0.527-1.648) | 0.808 |
| AFP(ng/ml) (≤20 vs.＞20) | 1.778 (0.942-3.353) | 0.076 |
| Tumor size（cm） | 1.082(0.993-1.178) | 0.072 |
| Tumor number (single vs. multiple) | 2.806 (1.549-5.083) | 0.001 |
| Vascular invasion (no vs. yes) | 2.544 (1.254-5.160) | 0.010 |
| Encapsulation (complete vs. no) | 1.533 (0.744-3.156) | 0.247 |
| MMP7 mRNA (low vs. high) | 1.499 (0.830-2.707) | 0.179 |

Multivariate analysis, Cox proportional hazards regression model

Variables were adopted for their prognostic significance by univariate analysis and no

obvious correlation between each other
