# Supplementary material for: Hypoxia-inducible factor-1 alpha, in association with inflammation, angiogenesis and MYC, is a critical prognostic factor in patients with HCC after surgery
Source: BMC Cancer. 2009 Dec 1;9:418. doi: 10.1186/1471-2407-9-418 (PMC2797816; doi:10.1186/1471-2407-9-418)
Supplement: Additional file 7 — Table S6: Multivariate analyses of variables associated with survival and recurrence including protein expression of HIF-1α as co-variable. (P = 0.021 for OS, P = 0.007 for DFS). [file 1471-2407-9-418-S7.DOC]

Table S5: **Multivariate analyses of variables associated with survival and recurrence including protein expression of HIF-1αas co-variable**

|  | Hazard ratio (95%CI) | *P* |
| --- | --- | --- |
| OS |  |  |
| AFP(ng/ml) (≤20 vs.＞20) | 1.132 (0.569-2.252) | 0.724 |
| γ-GT(U/I) (≤54 vs. ＞54) | 1.974 (0.992-3.931) | 0.053 |
| Tumor differentiation (Ⅰ+Ⅱ vs.Ⅲ+Ⅳ) | 1.638 (0.900 -2.981) | 0.106 |
| Tumor size（cm） | 1.126 (1.038-1.221) | 0.004 |
| Vascular invasion (no vs. yes) | 4.140 (1.893-9.054) | <0.001 |
| Encapsulation (complete vs. no) | 0.738 (0.365-1.492) | 0.398 |
| HIF-1α protein (low vs. high) | 2.108 (1.120-3.969) | 0.021 |
| DFS |  |  |
| Age (year) | 0.979 (0.953-1.005) | 0.119 |
| AFP(ng/ml) (≤20 vs.＞20) | 1.523 (0.797-2.910) | 0.203 |
| Tumor size（cm） | 1.112 (1.025-1.207) | 0.011 |
| Tumor number (single vs. multiple) | 2.816 (1.546-5.130) | 0.001 |
| Vascular invasion (no vs. yes) | 2.421 (1.204-4.868) | 0.013 |
| Encapsulation (complete vs. no) | 1.608 (0.798-3.240) | 0.184 |
| HIF-1α protein (low vs. high) | 2.265 (1.251-4.009) | 0.007 |

Multivariate analysis, Cox proportional hazards regression model

Variables were adopted for their prognostic significance by univariate analysis and no

obvious correlation between each other
