# Supplementary material for: Hypoxia-inducible factor-1 alpha, in association with inflammation, angiogenesis and MYC, is a critical prognostic factor in patients with HCC after surgery
Source: BMC Cancer. 2009 Dec 1;9:418. doi: 10.1186/1471-2407-9-418 (PMC2797816; doi:10.1186/1471-2407-9-418)
Supplement: Additional file 8 — Table S7: Multivariate analyses of variables associated with survival and recurrence including mRNA expression of VEGF as co-variable. (P = 0.081 for DFS). [file 1471-2407-9-418-S8.DOC]

**Table S6: Multivariate analyses of variables associated with survival and recurrence including mRNA expression of VEGF as co-variable.**

|  | Hazard ratio (95%CI) | *P* |
| --- | --- | --- |
| DFS |  |  |
| Age (year) | 0.930 (0.525-1.646) | 0.802 |
| AFP(ng/ml) (≤20 vs.＞20) | 2.193 (1.137-4.230) | 0.019 |
| Tumor size（cm） | 1.076 (0.988-1.171) | 0.093 |
| Tumor number (single vs. multiple) | 2.692 (1.483-4.889) | 0.001 |
| Vascular invasion (no vs. yes) | 2.418 (1.194-4.897) | 0.014 |
| Encapsulation (complete vs. no) | 1.471 (0.708-3.053) | 0.301 |
| VEGF mRNA (low vs. high) | 1.702 (0.936-3.094) | 0.081 |

Multivariate analysis, Cox proportional hazards regression model

Variables were adopted for their prognostic significance by univariate analysis and no

obvious correlation between each other
