# Supplementary material for: Hypoxia-inducible factor-1 alpha, in association with inflammation, angiogenesis and MYC, is a critical prognostic factor in patients with HCC after surgery
Source: BMC Cancer. 2009 Dec 1;9:418. doi: 10.1186/1471-2407-9-418 (PMC2797816; doi:10.1186/1471-2407-9-418)
Supplement: Additional file 9 — Table S2: Correlations between HIF-1α mRNA and protein expression. [file 1471-2407-9-418-S9.DOC]

Table S7: **Correlations between HIF-1α mRNA and protein expression.**

|  | | HIF-1α mRNA | | | | Total | |
| --- | --- | --- | --- | --- | --- | --- | --- |
|  |  | Low | | high | |  |  |
| HIF-1α protein | low | | 55 | | 16 | | 71 |
|  | high | | 17 | | 22 | | 39 |
| Total | | | 72 | | 38 | | 110 |

χ2 tests were used for the analysis of correlations (*P*=0.001).
